# Intake of omega-3 polyunsaturated fatty acids and fish associated with prevalence of low lean mass and muscle mass among older women: Analysis of Korea National Health and Nutrition Examination Survey, 2008-2011

**DOI:** 10.3389/fnut.2023.1119719

**Published:** 2023-02-21

**Authors:** Yeji Kim, Yongsoon Park

**Affiliations:** Department of Food and Nutrition, Hanyang University, Seoul, Republic of Korea

**Keywords:** low lean mass, muscle mass, sarcopenia, n-3 PUFA intake, fish intake, older women, KNHANES

## Abstract

The effects of dietary n-3 PUFA and fish on the risk of sarcopenia and muscle mass remain unclear. The present study investigated the hypothesis that intake of n-3 PUFA and fish is negatively associated with the prevalence of low lean mass (LLM) and positively correlated with muscle mass in older adults. Data from the Korea National Health and Nutrition Examination Survey, 2008-2011, 1,620 men and 2,192 women aged over 65 years were analyzed. LLM was defined as appendicular skeletal muscle mass divided by body mass index < 0.789 kg for men and <0.512 kg for women. Women and men with LLM consumed less eicosapentaenoic acid (EPA) docosahexaenoic acid (DHA) and fish. In women, but not men, the prevalence of LLM was associated with the intake of EPA and DHA (odds ratio, 0.65; 95% confidence interval, 0.48-0.90; *p* = 0.002) and fish (odds ratio, 0.59; 95% confidence interval, 0.42-0.82; *p* < 0.001). Muscle mass was also positively associated with the intake of EPA, DHA (*p* = 0.026), and fish (*p* = 0.005) in women, but not men. α-Linolenic acid intake was not associated with the prevalence of LLM and was not correlated with muscle mass. The findings suggest that consumption of EPA, DHA, and fish are negatively associated with the prevalence of LLM, and positively correlated with muscle mass in Korean older women, but not in older men.

## 1. Introduction

Sarcopenia, an age-associated loss of muscle mass and, strength, or performance is associated with increased adverse outcomes including falls, functional decline, frailty, and mortality, and has become a serious health issue among older adults (1). There are various complicated risk factors for sarcopenia, including aging, body composition, physical activity, comorbidities, and dietary intake (1). Malnutrition is well-known risk factor for sarcopenia, but the effect of individual nutrient such as protein, vitamin D, and n-3 polyunsaturated fatty acids (PUFA) on sarcopenia is unclear (2, 3). N-3 PUFA, eicosapentaenoic acid (EPA), and docosahexaenoic acid (DHA), which are abundant in fish, and α-linolenic acid (ALA), which is abundant in plants, have anti-inflammatory effects (4). It is becoming increasingly clear that inflammation processes play an important role in the pathogenesis of age-related sarcopenia (4). Low lean mass is one of the first factors to diagnose sarcopenia (1).

The risk of sarcopenia is negatively associated with the intake of total n-3 PUFA in kidney transplant patients (5) and serum levels of total n-3 PUFA in Korean older adults (6). Similarly, the ratio of daily intake of total n-3 PUFA to energy intake has been significantly associated with the risk of sarcopenic obesity in Korean older women, but not men, suggesting that n-3 PUFA have beneficial effects only in women (7). In kidney transplant patients, the intake of total n-3 PUFA have also been positively associated with muscle mass and negatively associated with the risk of low muscle mass (5).

Consistent with muscle mass, intakes of total n-3 PUFA, EPA, and DHA were positively associated with muscle function in American older adults (8), Japanese men (9), Finnish women (10), and Korean women (11). Sarcopenic older adults consumed less total n-3 PUFA and had lower erythrocyte EPA levels in the Maastricht Sarcopenia Study (12). Plasma levels of total n-3 PUFA, EPA, and DHA, as indicators of dietary intake of n-3 PUFA, were positively associated with muscle function among older adults in Europe (12–14), America (15), and Korea (6, 16). In addition, a meta-analysis of clinical trials found that supplementation with EPA and DHA increased muscle mass and muscle performance measured by timed up-and-go and gait speed in older adults (17). n-3 PUFA have been suggested to have anabolic and anti-catabolic properties in skeletal muscles by regulating the mammalian target of rapamycin (mTOR) signaling pathway and inflammatory factors (18).

Dietary intake of fatty fish was positively associated with grip strength in older adults in the Hertfordshire cohort study (19) and UK Biobank study (20). Similarly, adherence to the Mediterranean diet, which is known to contain abundant n-3 PUFA, was associated with better physical performance in postmenopausal women (21) and a lower risk of sarcopenia in Iranian older adults (22).

ALA intake was positively associated with muscle function measured by gait speed, one-leg stance, squat, Short Physical Performance Battery (SPPB), and grip strength, but not with muscle mass in older women in Finland (10). Supplementation with ALA increased muscle mass but not muscle function in older men but not in women (23).

To our knowledge, no study has shown an association between dietary intake of EPA and DHA, ALA, and fish and the prevalence of sarcopenia and muscle mass in older adults. Therefore, the purpose of the present study was to investigate the hypothesis that consumption of n-3 PUFA and fish is negatively associated with the prevalence of LLM and positively correlated with muscle mass in older men and women.

## 2. Methods

### 2.1. Participants

This study was based on data obtained from the Korea National Health and Nutrition Examination Survey (KNHANES) from 2008 to 2011. KNHANES was performed using a rolling sampling design involving a complex, stratified, multistage, probability-cluster survey of a representative sample of the non-institutionalized civilian population in South Korea (24). The survey was performed by the Korean Ministry of Health and Welfare and consisted of three components: a health interview survey, health examination survey, and nutrition survey. All participants signed an informed consent form (24). The study protocol was approved by the Institutional Review Board of Hanyang University (HYUIRB-202208-003).

**FIGURE 1:**
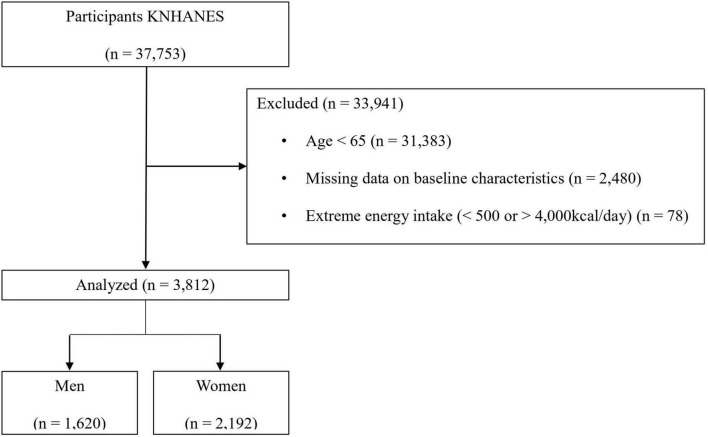
Flowchart of the inclusion and exclusion of participants.

Of the 37,753 participants, 33,941 were excluded for the following reasons: age 65 years or younger (*n* = 31,383); missing data on body mass index (BMI, kg/m^2^), appendicular skeletal muscle mass (ASM), and energy intake (*n* = 2,480); and extreme energy intake of less than 500 kcal/day or more than 4,000 kcal/day (*n* = 78). Finally, 1,620 men and 2,192 women were included in the present study (Figure 1).

### 2.2. Definition of low lean mass

Muscle mass was measured by dual-energy X-ray absorptiometry using a DISCOVERY-W fan-beam densitometer (Hologic, Marlborough, MA, USA). ASM (kg) was calculated as the sum of lean soft tissue in the bilateral upper and lower limbs. LLM was defined as < 0.789 kg of ASM/BMI in men and < 0.512 kg of ASM/BMI in women (25).

### 2.3. Study variables

Trained interviewers and medical staff assessed a wide range of covariates according to a standardized protocol (24). Anthropometry of waist circumference (WC) was measured at the midpoint between the inferior margin of the last rib and the iliac crest in the horizontal plane while the subject exhaled. Height and weight were measured to the nearest 0.1 cm and 0.1 kg, respectively, with participants wearing light clothing and being barefooted.

A questionnaire related to sociodemographic characteristics that included age, sex, smoking status, drinking status, regular exercise, living arrangement, and comorbidities was administered in the health interview. Smoking was defined as never (a person who has never smoked or has smoked less than five packs of cigarettes during their lifetime), former (a person who smoked more than five packs of cigarettes but who did not currently smoke), or current (a person who smokes more than five packs of cigarettes during their lifetime). Drinking was defined as the alcohol once or more times in a month. Regular exercise was defined as moderate exercise for 30 min, ≥ 5 times a week, vigorous exercise for 20 min, ≥ 3 times a week. Living arrangements were classified into two groups according to whether or not they live alone. Comorbidities were defined as participants with at least one medical history of hypertension, dyslipidemia, stroke, myocardial infarction, angina, osteoarthritis, rheumatoid arthritis, kidney failure, diabetes mellitus, or cancer.

Dietary intake data were assessed using a one-day 24-h dietary recall method during the household interview. Trained dietitians interviewed the participants to recall and describe all the foods and beverages they had consumed in the previous day. Fish was classified according to the Composition Table of Marine Products in Korea 2018 of the National Institute of Fisheries Science (26), and amount of n-3 PUFA in individual fish, as g/day was calculated based on the Food Composition Table developed by the Korea Rural Development Administration in 2018 (27).

### 2.4. Statistical analyses

Descriptive analysis was conducted using clustering and stratifying variables, using a survey procedure that applied individual weights to the analysis (28). Continuous variables were analyzed using the independent sample t-test and are expressed as the mean ± standard error of the mean. Categorical variables were analyzed using the chi-square test and are expressed as frequencies and percentages.

Multiple regression models were used to determine unsuitable potential covariates and examine the association between the prevalence of LLM and dietary intake of n-3 PUFA and fish after adjusting for potential covariates. In multivariate models, covariates with *p* < 0.20 were selected as confounding factors and included in the fully adjusted model (29). The participants were subdivided into three groups according to tertiles of dietary n-3 PUFA and fish intake, separately (30). Analysis of covariance (ANCOVA) with Bonferroni correction was performed to assess the mean differences in ASM/BMI among the intake tertile groups after adjustment for confounding variables. The relationship between LLM and dietary intake of n-3 PUFA and fish was analyzed using multivariable logistic regression analysis. This analysis was used to obtain odds ratios (ORs) and 95% confidence intervals (CIs) adjusted for confounding variables. *p*-values for linear trends were estimated using the median values within each tertile of dietary intake, considering the unequal distances between tertiles. *p* < 0.05 was considered statistically significant. Statistical analysis was performed using SPSS 27.0 software (SPSS Inc., Chicago, IL, USA).

## 3. Results

### 3.1. Baseline characteristics of participants

Compared to those without LLM, men and women with LLM were older, had higher BMI, greater WC, increased prevalence of obesity and abdominal obesity, more comorbidities, consumed less alcohol, and had reduced energy intake (Table 1). There were no statistically significant differences in the prevalence of LLM, smoking status, living alone, and ALA intake between the LLM and non-LLM groups. Women with LLM exercised less regularly and consumed less EPA, DHA, and fish than women without LLM. The total population with LLM was older, had higher BMI, greater WC, increased prevalence of obesity and abdominal obesity, more comorbidities, reduced exercise activities, and consumed less alcohol, energy, EPA, DHA, and fish than those without LLM (Supplementary Table 1).

**TABLE 1:** Baseline characteristics of men and women with and without low lean mass.

Variables	Men		*p*-Value*	Women		*p*-Value*
	Non-LLM (*n* = 1,167)	LLM (*n* = 453)		Non-LLM (*n* = 1,629)	LLM (*n* = 563)	
Age (year)	71.36 ± 0.13	73.04 ± 0.22	<0.001	71.81 ± 0.12	73.01 ± 0.19	<0.001
BMI (kg/m^2^)	22.62 ± 0.08	24.29 ± 0.14	<0.001	23.46 ± 0.08	25.96 ± 0.14	<0.001
Obesity, *n* (%)	222 (19.0)	171 (37.7)	<0.001	495 (30.4)	335 (59.5)	<0.001
WC (cm)	83.46 ± 0.26	87.60 ± 0.42	<0.001	81.83 ± 0.23	87.10 ± 0.40	<0.001
Abdominal obesity, *n* (%)	285 (24.5)	185 (40.9)	<0.001	602 (37.1)	329 (58.9)	<0.001
Smoking status, *n* (%)			0.069			0.054
Never	200 (17.3)	74 (16.7)		1,446 (90.1)	501 (90.6)	
Former	646 (56.0)	275 (61.9)		69 (4.3)	31 (5.6)	
Current	308 (26.7)	95 (21.4)		90 (5.6)	21 (3.8)	
Drinking status, *n* (%)	684 (59.3)	224 (50.5)	0.005	279 (17.4)	72 (13.0)	0.037
Regular exercise, *n* (%)	260 (22.3)	88 (19.4)	0.305	322 (19.8)	68 (12.1)	<0.001
Living alone, *n* (%)	80 (6.9)	30 (6.7)	0.957	418 (25.7)	142 (25.3)	0.214
Comorbidities	720 (62.1)	350 (78.5)	< 0.001	1,289 (80.1)	493 (88.4)	<0.001
**Dietary intake**						
Energy intake (kcal/day)	1,897.07 ± 17.56	1,710.18 ± 26.21	< 0.001	1,459.16 ± 12.41	1,365.54 ± 19.94	<0.001
EPA+DHA (g/day)	0.84 ± 0.04	0.76 ± 0.06	0.830	0.50 ± 0.02	0.40 ± 0.03	0.002
ALA (g/day)	1.47 ± 0.06	1.24 ± 0.08	0.393	1.14 ± 0.07	1.01 ± 0.06	0.088
Fish (g/day)	43.56 ± 2.21	36.83 ± 3.03	0.545	26.44 ± 1.37	18.65 ± 1.74	<0.001

Values are expressed as mean ± standard error of the mean for continuous variables or as number (percentage) for categorical variables. *p-values were calculated using independent sample t-test for continuous variables; chi-square test for categorical variables LLM, low lean mass; BMI, body mass index; WC, waist circumference; PUFA, polyunsaturated fatty acid; EPA, Eicosapentaenoic acid; DHA, Docosahexaenoic acid; ALA, alpha linolenic acid.

### 3.2. Associations between prevalence of LLM and intakes of n-3 PUFA and fish

Logistic regression analysis revealed that the prevalence of LLM was negatively associated with the intake of EPA and DHA, and fish, but not ALA in women, after adjusting for potential confounders (Table 2). However, there was no significant association between the prevalence of LLM and the intake of n-3 PUFA and fish in men. After full adjustment, the intake of EPA and DHA and fish were also negatively associated in the total study population (Supplementary Table 2). We divided women into 4 groups; non-sarcopenic non-obesity, sarcopenic non-obesity, non-sarcopenic obesity, and sarcopenic obesity. The prevalence of LLM was significantly associated with intake of EPA and DHA, and fish in women with non-sarcopenic non-obesity, sarcopenic non-obesity, and sarcopenic obesity (Supplementary Table 3).

**TABLE 2:** Associations between prevalence of low lean mass and n-3 PUFA and fish intake in men and women.

Variables	Tertiles of n-3 PUFA and fish intake			*p*-Trend
	T1	T2	T3	
**Men**				
EPA+DHA (g/day), range	<0.13	0.13 ≤ to < 0.67	≥0.67	0.522
No. with/without LLM	155/385	168/372	130/410	
OR (95% CI)	1	1.214 (0.881 – 1.673)	0.962 (0.673– 1.376)	
ALA (g/day), range	< 0.58	0.58 ≤ to < 1.30	≥ 1.30	0.404
No. with/without LLM	175/365	156/384	122/418	
OR (95% CI)	1	0.897 (0.657 – 1.225)	0.842 (0.582 – 1.217)	
Fish (g/day), range	< 0.24	0.24 ≤ to < 34.20	≥ 34.20	0.567
No. with/without LLM	152/388	169/371	132/408	
OR (95% CI)	1	1.375 (0.983 – 1.922)	1.038 (0.748 – 1.442)	
**Women**				
EPA+DHA (g/day), range	< 0.06	0.06 ≤ to < 0.40	≥ 0.40	0.002
No. with/without LLM	207/523	195/536	161/570	
OR (95% CI)	1	1.088 (0.818 – 1.447)	0.654 (0.478–0.896)	
ALA (g/day), range	< 0.43	0.43 ≤ to < 0.95	≥ 0.95	0.602
No. with/without LLM	189/541	194/537	180/551	
OR (95% CI)	1	1.272 (0.940 – 1.722)	1.151 (0.829–1.597)	
Fish (g/day), range	< 0.00	0.00 ≤ to < 15.33	≥ 15.33	<0.001
No. with/without LLM	239/626	165/431	159/572	
OR (95% CI)	1	1.214 (0.926 – 1.592)	0.590 (0.423–0.823)	

Odds ratios (ORs) and 95% confidence intervals (CIs) were presented. The logistic regression model was adjusted for age, abdominal obesity, alcohol consumption, comorbidities, and energy intake for men and for age, abdominal obesity, smoking, regular exercise, and energy intake for women. LLM, low lean mass; PUFA, polyunsaturated fatty acid; EPA, Eicosapentaenoic acid; DHA, Docosahexaenoic acid; ALA, alpha linolenic acid.

### 3.3. Associations between muscle mass and intakes of dietary n-3 PUFA and fish

After adjusting for potential confounders, ANCOVA revealed a significant positive association between muscle mass and intake of EPA, DHA, and fish, but not ALA in women, as a continuous and non-continuous variable (Table 3). However, there were no associations between muscle mass and intakes of n-3 PUFA and fish in men (Table 3) and the total population (Supplementary Table 4).

**TABLE 3:** Correlation between muscle mass and n-3 PUFA and fish intake in men and women.

Variables (g/day)		Tertiles of n-3 PUFA and fish intake			*p*-Trend*	Continuous	
		**T1**	**T2**	**T3**		***r***	***p*-Value**
Men	EPA+DHA, range	< 0.13	0.13 ≤ to < 0.67	≥ 0.67			
	ASM/BMI	0.85 ± 0.004	0.84 ± 0.004	0.85 ± 0.004	0.743	0.012	0.689
	ALA, range	< 0.58	0.58 ≤ to < 1.30	≥ 1.30			
	ASM/BMI	0.84 ± 0.004	0.84 ± 0.004	0.86 ± 0.004	0.754	0.017	0.603
	Fish, range	< 0.24	0.24 ≤ to < 34.20	≥ 34.20			
	ASM/BMI	0.85 ± 0.004	0.83 ± 0.004	0.85 ± 0.004	0.520	0.027	0.316
Women	EPA+DHA, range	< 0.06	0.06 ≤ to < 0.40	≥ 0.40			
	ASM/BMI	0.56 ± 0.003	0.56 ± 0.003	0.57 ± 0.003	0.026	0.054	0.013
	ALA, range	< 0.43	0.43 ≤ to < 0.95	≥ 0.95			
	ASM/BMI	0.56 ± 0.003	0.56 ± 0.003	0.57 ± 0.003	0.445	<0.001	0.964
	Fish, range	< 0.00	0.00 ≤ to < 15.33	≥ 15.33			
	ASM/BMI	0.55 ± 0.003	0.56 ± 0.003	0.57 ± 0.003	0.005	0.083	<0.001

*p-Trend for the differences in muscle mass (ASM/BMI) according to tertiles of n-3 PUFA and seafood intake after adjustment for confounding factors, including age, abdominal obesity, drinking, comorbidities, and energy intake for men and age, abdominal obesity, smoking, regular exercise, and energy intake for women using ANCOVA test with Bonferroni correction. Values represent correlations (r). PUFA, polyunsaturated fatty acid; EPA, Eicosapentaenoic acid; DHA, Docosahexaenoic acid; ALA, alpha linolenic acid.

## 4. Discussion

The present study demonstrates that consumption of EPA, DHA, and fish were negatively associated with the prevalence of LLM and positively correlated with muscle mass in Korean older women, but not in men, after adjusting for potential confounders in KNHANES data from 2008 to 2011. Consistent with the present study, a higher total n-3 PUFA intake was associated with a lower risk of sarcopenia among kidney transplant patients in Brazil (5). The risk of sarcopenia was also negatively associated with serum levels of total n-3 PUFA and decreased by 71% with each standard deviation increment of serum level of total n-3 PUFA in Korean older adults (6). Yang et al. (7) also showed that the ratio of daily total n-3 PUFA intake to energy intake was negatively associated with the risk of sarcopenic obesity in older Korean women, but not in men.

Dos Reis et al. (5) observed that the intake of total n-3 PUFA was positively associated with muscle mass, estimated by ASM divided by the height squared, in kidney transplant patients. The Maastricht Sarcopenia Study of a community-dwelling population found that older adults with sarcopenia had a significantly lower intake of total n-3 PUFA and lower erythrocyte EPA levels than those without sarcopenia (12). A meta-analysis of clinical trials reported that supplementation with EPA and DHA elicited an approximately 0.33 kg increase in muscle mass in older adults, especially when more than 2 g/day of EPA and DHA was administered (17). n-3 PUFA increases the rate of muscle protein synthesis by stimulating the mTOR signaling pathway, and prevents muscle degradation by reducing inflammatory signaling pathway signaling (18).

Epidemiologic studies have consistently reported that the intake of total n-3 PUFA, EPA, and DHA was associated with muscle function measured by leg strength, time to rise from a chair, timed up-and-go, gait speed, handgrip strength, and SPPB in American older adults (8), Japanese men (9), Finnish women (10), and Korean women (11). Similarly, high plasma concentrations of total n-3 PUFA, EPA, and DHA were associated with gait speed, handgrip strength, and SPPB among older adults in a Three-City study (13) as well as in Korea (6), United States (15), and Italy (14). In particular, our previous study showed that erythrocyte EPA and DHA levels are associated with gait speed in Korean older adults (16). A meta-analysis of clinical trials found that supplementation with EPA and DHA improved gait speed in older adults (17). However, Rossato et al. (31) reported that intake of EPA and DHA was not associated with muscle strength or voluntary peak isokinetic knee extensor strength in American older adults, and their average intake of EPA and DHA was 0.1 g/day. On the other hand, the average intake of EPA and DHA was 0.6 g/day among Korean older adults in the present study, which was six times higher than that in Americans. The Food and Agriculture Organization of the United Nations reported that the intake of aquatic food, a rich source of n-3 PUFA, was more than 50 kg per capita per year among Koreans, the highest in the world, and only 20-30 kg per capita per year among Americans (32).

To our knowledge, no study has investigated the association between fish intake and muscle mass. However, previous studies have reported that fish intake is positively associated with muscle function. Muscle function measured by grip strength was positively associated with consumption of fatty fish and oily fish, but not white fish and shells, in older adults from the Hertfordshire cohort (19) and from the UK Biobank study (20). In addition, a Mediterranean diet, known to contain many fish, was negatively associated with the risk of sarcopenia and positively associated with gait speed in older Iranian adults (22). The Finnish Osteoporosis Risk Factor and Prevention Fracture Prevention Study also found that the Mediterranean diet score was significantly associated with gait speed in older women (21). Rondanelli et al. (33) suggested that fish contain anti-sarcopenic compounds, such as n-3 PUFA, proteins, vitamin D, magnesium, and carnitine, which could reduce inflammation and improve muscle response to exercise and diet.

The Osteoporosis Risk Factor and Prevention Fracture Prevention Study reported that ALA intake was positively associated with muscle function, but not muscle mass, among Finnish older women (10). The Maastricht Sarcopenia Study analysis found that ALA intake was significantly associated with muscle function, but erythrocyte levels of ALA, a marker for dietary intake, were not associated with muscle function in Dutch older adults (12). In addition, supplementation of 14 g/day of ALA with a resistance training program increased knee flexor muscle thickness and had an effect on muscle functions, such as chest and leg press, in Canadian older men (23). However, the increased muscle thickness might not be due to the intake of ALA but to exercise, since all participants in the trial were on resistance training programs (23). Thus, the documented effect of ALA intake on muscle function is inconsistent and might not be associated with muscle mass, which supports the results of the present study.

A noteworthy point of our findings was that intake of EPA, DHA, and fish was associated with the prevalence of LLM and muscle mass in older women, but not in older men. Consistent with the present study, a higher ratio of daily total n-3 PUFA intake to energy intake was negatively associated with the risk of sarcopenic obesity (7), and intake of EPA and DHA was positively associated with handgrip strength (11) in Korean older women, but not in men, suggesting that intake of n-3 PUFA might be beneficial for sarcopenia among Korean women. In the present study, intakes of EPA and DHA, and ALA were analyzed separately instead of n-3 PUFA, and appendicular skeletal muscle mass was evaluated instead of handgrip strength. Asian people, especially Asian women, tend to have lower muscle mass and higher body fat mass with central adiposity than Western populations (34, 35). In the present study, the average BMI and WC of sarcopenic women were 26 kg/m^2^ and 87 cm, respectively, indicating that sarcopenic women were mostly abdominal obese, but sarcopenic men were not. Thus, in the present study, muscle mass was calculated based on ASM divided by BMI, but not by height squared (m^2^), to consider sarcopenic obesity. Previous epidemiological studies have shown that patients with sarcopenic obesity have higher levels of cytokines, such as C-reactive protein (CRP), interleukin-6 (IL-6), and monocyte chemotactic protein-1 than those with sarcopenia (36–38). Inflammation is a well-known component of the pathophysiology of muscle wasting (39). A meta-analysis of clinical trials showed that n-3 PUFA supplementation reduced inflammatory biomarkers such as CRP and IL-6 in older adults (40). Al-Safi et al. (41) further reported that supplementation with EPA and DHA decreased the levels of IL-1 and tumor necrosis factor-alpha (TNF-α), and the reduction in cytokines was greater among obese women than among normal-weight women, suggesting that n-3 PUFA could be more beneficial for those with inflammation. Additionally, women in general have a greater capacity to convert ALA to DHA than men due to estrogen; thus, the plasma level of DHA is higher in women than in men (42). Canon et al. (43) reported that estrogen decreases the levels of CRP and IL-6 and inhibits chronic inflammation. Furthermore, Smith et al. (44) observed that supplementation of EPA and DHA enhanced muscle anabolism when plasma leucine concentrations were clamped at 165-175 μmol/L in healthy adults. However, McGlory et al. (45) found that supplementation with EPA and DHA had no effect on muscle protein synthesis during peak plasma leucine concentrations of 250-300 μmol/L achieved by the ingestion of 30 g of whey protein in young men. It is possible that ingestion of 30 g of whey protein could maximize the rate of muscle protein synthesis to the extent that fish oil supplementation would not have exerted a further anabolic influence (46, 47). Thus, previous studies have suggested that a beneficial effect of n-3 PUFA on muscle synthesis might be observed when protein is insufficient (44–47). In the present study, the average protein intake was 62 g/day in men aged 65 years or older, similar to the Korean dietary reference intake (KDRIs) of 60 g/day, but the average protein intake was 45 g/day in women aged 65 years or older, which is lower than the KDRIs of 50 g/day (48, 49). Additionally, protein intake was lower among older men with LLM (57 g/day vs. 63 g/day; *p* = 0.002) and women with LLM (43 g/day vs. 45 g/day; *p* = 0.015) than those without LLM, although protein intake as g/kcal/day was not different between men with and without LLM (33 mg/kcal vs. 33 mg/kcal; *p* = 0.573) and between women with and without LLM (31 mg/kcal vs. 31 mg/kcal; *p* = 0.460). As Korean women consume insufficient amounts of protein, the beneficial effect of n-3 PUFA on LLM might be observed only in Korean women.

The major strength of the present study was that the data were gathered from a nationally representative survey throughout Korea; thus, the findings can be generalized to older adults in Korea. However, some limitations should be considered when interpreting the results of this study. First, the cross-sectional study design was unable to establish a causal relationship between the prevalence of LLM and the intake of n-3 PUFA and fish. Second, muscle strength or performance were not measured in the KNHANES 2008-2011, and sarcopenia could not be diagnosed. However, loss of muscle mass with aging is clinically important because it leads to diminished strength and exercise capacity (50). Third, the dietary intake of one day was assessed using the 24-h recall method, which could have recall bias and did not reflect the usual dietary intake.

## 5. Conclusion

The present study demonstrates that consumption of high levels of EPA, DHA, and fish could have beneficial effects on the prevention of LLM by improving muscle mass in older women. Further studies are needed to verify the preventive effects of EPA, DHA, and fish consumption on sarcopenia in large population-based longitudinal studies of diverse ethnic origins.

## Data availability statement

The datasets presented in this study can be found in online repositories. The names of the repository/repositories and accession number(s) can be found below: https://knhanes.kdca.go.kr/knhanes/sub03/sub03_01.do.

## Ethics statement

The studies involving human participants were reviewed and approved by Institutional Review Board of Hanyang University (HYUIRB-202208-003). The patients/participants provided their written informed consent to participate in this study.

## Author contributions

YK performed statistical analyses and wrote the manuscript. YP designed the study, revised the manuscript, and was responsible for this work. Both authors have read and agreed to the published version of the manuscript.
