# Supplementary material for: Intake of omega-3 polyunsaturated fatty acids and fish associated with prevalence of low lean mass and muscle mass among older women: Analysis of Korea National Health and Nutrition Examination Survey, 2008-2011
Source: Front Nutr. 2023 Feb 21;10:1119719. doi: 10.3389/fnut.2023.1119719 (PMC9989170; doi:10.3389/fnut.2023.1119719)
Supplement: Supplementary file 1 [file Data_Sheet_1.docx]

Supplementary Material

Intake of omega-3 polyunsaturated fatty acids and fish associated with prevalence of low lean mass and muscle mass among older women: Analysis of Korea National Health and Nutrition Examination Survey, 2008-2011

Yeji Kim, MS, Yongsoon Park, PhD*

*** Correspondence:** Yongsoon Park, PhD: yongsoon@hanyang.ac.kr

# Supplementary Tables

**Supplementary Table 1.** Baseline characteristics of the study population with and without low lean mass

| Variables | Total population | | *p*-Value^*^ |
| --- | --- | --- | --- |
|  | Non-LLM (n = 2,796) | LLM (n = 1,016) |  |
| Age (year) | 71.62 ± 0.09 | 73.02 ± 0.15 | <0.001 |
| Sex, men | 1,167 (41.7) | 453 (44.6) | 0.493 |
| BMI (kg/m^2^) | 23.11 ± 0.06 | 25.21 ± 0.10 | <0.001 |
| Obesity, n (%) | 717 (25.6) | 506 (49.8) | <0.001 |
| WC (cm) | 82.51 ± 0.18 | 87.32 ± 0.29 | <0.001 |
| Abdominal obesity, n (%) | 887 (31.9) | 514 (50.8) | <0.001 |
| Smoking status, n (%) |  |  | 0.069 |
| Never | 1,646 (59.7) | 575 (57.7) |  |
| Former | 715 (25.9) | 306 (30.7) |  |
| Current | 398 (14.4) | 116 (11.6) |  |
| Drinking status, n (%) | 963 (35.0) | 296 (29.7) | 0.004 |
| Regular exercise, n (%) | 582 (20.8) | 156 (15.4) | <0.001 |
| Living alone, n (%) | 498 (17.8) | 172 (17.0) | 0.337 |
| Comorbidities | 2,009 (72.6) | 843 (84.0) | <0.001 |
| Dietary intake |  |  |  |
| Energy intake (kcal/day) | 1,641.94 ± 11.07 | 1,519.20 ± 16.95 | <0.001 |
| EPA+DHA (g/day) | 0.64 ± 0.02 | 0.56 ± 0.03 | 0.016 |
| ALA (g/day) | 1.28 ± 0.05 | 1.11 ± 0.05 | 0.100 |
| Fish (g/day) | 33.59 ± 1.23 | 26.76 ± 1.68 | 0.006 |

Values are expressed as the mean ± standard error of the mean for continuous variables or as the number (percentage) for categorical variables

^*^*p*-values were calculated using independent sample t-test for continuous variables; chi-square test for categorical variables

LLM, low lean mass; *BMI, body mass index; WC,* *waist circumference; PUFA, polyunsaturated fatty acid; EPA, Eicosapentaenoic acid; DHA, Docosahexaenoic acid; ALA,* *alpha linolenic acid*

**Supplementary Table 2.** Associations between prevalence of low lean mass and n-3 PUFA and fish intake in the study population

| Variables | Tertiles of n-3 PUFA and fish intake | | | *p*-Trend |
| --- | --- | --- | --- | --- |
|  | T1 | T2 | T3 |  |
| Total population |  |  |  |  |
| EPA+DHA (g/day), range | < 0.08 | 0.08 ≤ to < 0.50 | ≥ 0.50 | 0.004 |
| No. with/without LLM | 369/901 | 361/910 | 286/985 |  |
| OR (95% CI) | 1 | 1.058 (0.830 – 1.348) | 0.734 (0.570 – 0.945) |  |
| ALA (g/day), range | < 0.48 | 0.48 ≤ to < 1.07 | ≥ 1.07 | 0.989 |
| No. with/without LLM | 361/909 | 344/927 | 311/960 |  |
| OR (95% CI) | 1 | 1.012 (0.798 – 1.284) | 1.008 (0.777 – 1.306) |  |
| Fish (g/day), range | < 0.00 | 0.00 ≤ to < 21.87 | ≥ 21.87 | 0.008 |
| No. with/without LLM | 391/1,010 | 335/805 | 290/981 |  |
| OR (95% CI) | 1 | 1.201 (0.959 – 1.505) | 0.799 (0.632 – 0.976) |  |

Odds ratios (ORs) and 95% confidence intervals (CIs) are presented. The logistic regression model was adjusted for age, sex, abdominal obesity, smoking, drinking, regular exercise, and energy intake for total population

LLM, low lean mass; *PUFA, polyunsaturated fatty acid; EPA, Eicosapentaenoic acid; DHA, Docosahexaenoic acid; ALA,* *alpha linolenic acid*

**Supplementary Table 3.** Associations between prevalence of low lean mass and n-3 PUFA and fish intake in women with non-sarcopenic non-obesity, sarcopenic non-obesity, non-sarcopenic obesity, and sarcopenic obesity

| Variables | Tertiles of n-3 PUFA and fish intake | | | *p*-Trend^*^ |
| --- | --- | --- | --- | --- |
|  | T1 | T2 | T3 |  |
| Non-sarcopenic non-obesity |  |  |  |  |
| EPA+DHA (g/day), range | < 0.06 | 0.06 ≤ to < 0.40 | ≥ 0.40 | 0.044 |
| No. with/without LLM | 360/360 | 411/373 | 363/325 |  |
| OR (95% CI) | 1 | 1.037 (0.753 – 1.428) | 1.376 (0.966 – 1.961) |  |
| Fish (g/day), range | < 0.00 | 0.00 ≤ to < 15.33 | ≥ 15.33 | 0.010 |
| No. with/without LLM | 439/426 | 348/335 | 347/297 |  |
| OR (95% CI) | 1 | 0.873 (0.627 – 1.216) | 1.426 (1.005 – 2.024) |  |
| Sarcopenic non-obesity |  |  |  |  |
| EPA+DHA (g/day), range | < 0.06 | 0.06 ≤ to < 0.40 | ≥ 0.40 | 0.003 |
| No. with/without LLM | 85/635 | 90/694 | 53/635 |  |
| OR (95% CI) | 1 | 1.168 (0.774 – 1.763) | 0.574 (0.366 – 0.901) |  |
| Fish (g/day), range | < 0.00 | 0.00 ≤ to < 15.33 | ≥ 15.33 | 0.015 |
| No. with/without LLM | 86/779 | 93/590 | 49/595 |  |
| OR (95% CI) | 1 | 1.611 (1.069 – 2.428) | 0.690 (0.423 – 1.125) |  |
| Non-sarcopenic obesity |  |  |  |  |
| EPA+DHA (g/day), range | < 0.06 | 0.06 ≤ to < 0.40 | ≥ 0.40 | 0.287 |
| No. with/without LLM | 154/566 | 163/621 | 178/510 |  |
| OR (95% CI) | 1 | 0.880 (0.632 – 1.227) | 1.142 (0.790 – 1.651) |  |
| Fish (g/day), range | < 0.00 | 0.00 ≤ to < 15.33 | ≥ 15.33 | 0.177 |
| No. with/without LLM | 187/678 | 138/545 | 170/474 |  |
| OR (95% CI) | 1 | 0.933 (0.634 – 1.373) | 1.220 (0.853 – 1.743) |  |
| Sarcopenic obesity |  |  |  |  |
| EPA+DHA (g/day), range | < 0.06 | 0.06 ≤ to < 0.40 | ≥ 0.40 | 0.034 |
| No. with/without LLM | 121/599 | 120/664 | 94/594 |  |
| OR (95% CI) | 1 | 1.010 (0.701 – 1.455) | 0.771 (0.515 – 1.156) |  |
| Fish (g/day), range | < 0.00 | 0.00 ≤ to < 15.33 | ≥ 15.33 | 0.006 |
| No. with/without LLM | 153/712 | 104/579 | 78/566 |  |
| OR (95% CI) | 1 | 0.904 (0.640 – 1.276) | 0.576 (0.388 – 0.855) |  |

^*^*p*-Trend for the logistic regression model adjusting for age, abdominal obesity, smoking, regular exercise, and energy intake for women

**Supplementary Table 4.** Correlation between muscle mass and n-3 PUFA and fish intake in the study population

| Variables (g/day) | Tertiles of n-3 PUFA and fish intake | | | *p*-Trend^*^ | Continuous | |
| --- | --- | --- | --- | --- | --- | --- |
|  | T1 | T2 | T3 |  | *r* | *p*-Value |
| Total population |  |  |  |  |  |  |
| EPA+DHA, range | < 0.08 | 0.08 ≤ to < 0.50 | ≥ 0.50 |  |  |  |
| ASM/BMI | 0.66 ± 0.005 | 0.67 ± 0.004 | 0.71 ± 0.005 | 0.152 | 0.162 | <0.001 |
| ALA, range | < 0.48 | 0.48 ≤ to < 1.07 | ≥ 1.07 |  |  |  |
| ASM/BMI | 0.66 ± 0.004 | 0.68 ± 0.005 | 0.71 ± 0.005 | 0.331 | 0.067 | 0.094 |
| Fish, range | < 0.00 | 0.00 ≤ to < 21.87 | ≥ 21.87 |  |  |  |
| ASM/BMI | 0.66 ± 0.004 | 0.67 ± 0.005 | 0.72 ± 0.005 | 0.127 | 0.147 | <0.001 |

^*^*p*-Trend for the differences in muscle mass (ASM/BMI) according to tertiles of n-3 PUFA and seafood intakes after adjustment for confounding factors; the covariates were age, sex, abdominal obesity, smoking, drinking, regular exercise, and energy intake for total population using ANCOVA test with Bonferroni correction

Values represent correlations (r)

*PUFA, polyunsaturated fatty acid; EPA, Eicosapentaenoic acid; DHA, Docosahexaenoic acid; ALA,* *alpha linolenic acid*
